# The neuroprotective effect of BSA-based nanocurcumin against 6-OHDA-induced cell death in SH-SY5Y cells

**Published:** 2019

**Authors:** Roksana Sookhaklari, Bita Geramizadeh, Morteza Abkar, Maryam Moosavi

**Affiliations:** 1 *Students Research Committee, School of Medicine, Shiraz University of Medical Sciences, Shiraz, Iran*; 2 *Department of Pathology, Transplant Research Center, Shiraz University of Medical Sciences, Shiraz, Iran*; 3 *Nanobiology and Nanomedicine Research Centre, Shiraz University of Medical sciences, Shiraz, Iran*; 4 *Shiraz Neuroscience Research Centre, Shiraz University of Medical sciences, Shiraz, Iran*

**Keywords:** 6-OHDA, SH-SY5Y, Nanocurcumin, Akt, Parkinson’s disease

## Abstract

**Objective:**

Parkinson’s disease (PD) is regarded as the second most common neurodegenerative disease affecting elderly population. There is a tendency toward finding natural cures to suppress the initiation and progression of this disease. Some epidemiological studies indicated lower incidence of PD in populations that consume curry. Curcumin, as the main ingredient of turmeric, has been supposed to have a protective role against PD progression. However, low bioavailability of curcumin is still a challenge in evaluation of the therapeutic potential of this substance. In this study, we aimed to produce a BSA-based nanocurcumin to assess its effect on 6-hydroxydopamine (6-OHDA)-induced death and Akt signaling disruption in SH-SY5Y cells.

**Materials and Methods:**

BSA-based nanocurcumin was produced using desolvation method. Human neuroblastoma cells were treated with OHDA with/without different doses of nanocurcumin and MTT test was used to assess their viability besides observing cells morphological changes. The protective doses of nanocurcumine were chosen according to MTT results and western blot studies were done to assess p-Akt/t-Akt ratio.

**Results:**

6-OHDA exposure led to decreased cell viability, while nanocurcumin at doses of 400 and 500 nM prevented cell death. Moreover, this nanoformulation of curcumin restored p-Akt/t-Akt decrement induced by 6-OHDA. The protective effect of BSA-based nanocurcumin was estimated to be at least 4 time higher than that of natural curcumin according to the MTT results.

**Conclusion:**

It seems that BSA-based nanocurcumin can be regarded as a potent substitute for natural curcumin in protecting SH-SY5Y cell as a cellular model of PD.

## Introduction

Increase in the aging population in many countries is associated with the increment of the prevalence of the second most common neurodegenerative disease, Parkinson’s disease (PD) (Blesa and Przedborski, 2014 ▶; Cai et al., 2015 ▶). This disease is a multifactorial neurological disorder characterized by loss of dopaminergic neurons and subsequent loss of dopamine in the midbrain region (Shin et al., 2013 ▶). 

Curcumin is the active substance of turmeric which is frequently used in cuisine in many countries (Prasad et al., 2014 ▶). 

A wide range of studies has shown curcumin’s pharmacological benefits like antioxidant, antimicrobial, anti-inflammatory, anti-aging, anti-Alzheimer’s disease, anti-Parkinson’s disease, and anticancer activities (Nair et al., 2015 ▶; Young et al., 2014 ▶). However, its low solubility in water and insufficient oral bioavailability have restricted its use in clinical trials (Tsai et al., 2011a ▶; Young et al., 2014 ▶). Using nanotechnology and breaking this compound to nano level, higher bioavailability and improved therapeutic effect on the brain, could be achieved (Mourtas et al., 2014 ▶). Many studies have reported that breaking curcumin to nanosize, increases its bioavailability and therapeutic efficiency in many diseases (Cheng et al., 2013 ▶; Mourtas et al., 2014 ▶; Pan et al., 1999 ▶; Takahashi et al., 2009 ▶; Tsai et al., 2011a ▶; Vandita et al., 2012 ▶). In a PD model of flies, nanocurcumin acted effectively in decreasing PD symptoms (Siddique et al., 2014 ▶). The bioavailability of nanocurcumin was found higher than curcumin in mice brain and showed a protective effect against oxidative stress in mice brain (Nazari et al., 2014 ▶). Nanocurcumin has also been shown to have a higher mean residential time in mice brain comparing to natural curcumin (Cheng et al., 2013 ▶).

Some toxins like as MPTP (1-methyl-4-phenyl-1,2,3,6-tetrahydropyridine), rotenone, 6-OHDA (6-hydroxydopamine) and paraquat are routinely applied to induce the experimental models of PD (Bove et al., 2005 ▶). 6-OHDA is structurally identical to dopamine, yet its hydroxyl group leads to the toxicity of this substance in dopaminergic cells; thus, it is regarded as an attractive agent to induce PD. 6-OHDA penetrates the cell via a dopamine reuptake transporter (Ljungdahl et al., 1971 ▶) and leads to cell death (Blum et al., 2000 ▶).

The aim of the present study was to formulate nano-sized curcumin and evaluate its potential protective effect against 6-OHDA-induced SH-SY5Y cell death (as a cellular model of PD (Song et al., 2012 ▶). Moreover, as some reports showed the role of protein kinase B (PKB), known as Akt, in 6-OHDA toxicity (Amiri et al., 2016 ▶; Chen et al., 2004 ▶), the expression level of this protein was assessed by western blotting.

## Materials and Methods

**Materials**

SH-SY5Y cells were purchased from Pasteur institute, Iran. Cell culture materials including DMEM/F12, Fetal Bovine Serum (FBS) and penicillin-streptomycin were obtained from Gibco life technologies. Curcumin and 6-OHDA were purchased from Sigma-Aldrich. Western blot antibodies including beta-actin, phospho-Akt (Ser473), total Akt (tAkt) and secondary HRP-conjugated were purchased from Cell Signaling Technology. Amersham ECL select reagent kit was purchased from GE health care and PVDF membrane was purchased from Millipore. Other reagents were obtained from usual commercial sources.

**Preparation of BSA-based nanocurcumin**

Curcumin nanoparticles were prepared using bovine serum albumin as a carrier. The protocol used, was a previously published method with some modifications (Aniesrani Delfiya et al., 2016 ▶; Jithan et al., 2011 ▶). Briefly, a 3% BSA solution was prepared by dissolving a desired amount of lyophilized BSA powder in distilled water. Curcumin was separately dissolved in acetone at a concentration of 2.5%. Subsequently, dissolved curcumin was added intermittently into the BSA solution on a magnetic stirrer at 500 rpm at room temperature until the solution became turbid. After that, 110 µL of 8 % glutaraldehyde (in distilled water) was added to the solution to achieve the cross-linking of the BSA nanoparticles. This process was continued on the stirrer for a period of 24 hr at 4 ^o^C. Then, the suspension was purified by 5 cycles of differential centrifugation (at 3000 rpm for 30 min), and the pellet was dispersed into a desired volume of distilled water (final volume of 10 mL). The dispersed nanoparticles were finally lyophilized and store at 4 ^o^C. The formation of nanoparticles was assessed by a scanning electron microscope.

**Characterization of antigen-loaded nanoparticles**

Scanning electron microscopy (SEM) was performed using a TESCAN Mira3-XMU (Czech Republic) microscope by dropping a dilute suspension of the nanoparticles on a glass slide.

**Human neuroblastoma Cell Culture**

Human SH-SY5Y cells were grown in DMEM/F12 media supplemented with 10% fetal bovine serum, 100 U /ml penicillin and 100 µg/ml streptomycin. The cells were seeded in 96-well plates to assess cell viability via MTT (3-[4, 5-dimethylthiazol-2-yl]-2, 5-diphenyl tetrazolium bromide) test. For western blot studies, 6-well plates were used. The cells were incubated at 37°C in 90% humidified atmosphere with 5% CO_2_. As some reports indicated that differentiated SH-SY5Y cells have some changes in Akt signaling which lead to high tolerance against 6-OHDA (Cheung et al., 2009 ▶), undifferentiated SH-SY5Y cells were proposed as more appropriate ones to study neurotoxicity or neuroprotection in experimental PD studies (Schule et al., 2009 ▶)***. ***Therefore, in this research, undifferentiated SH-SY5Y cells were used.

**Treatment**

A pilot study was done to choose the effective dose of nanocurcumin. Accordingly, the doses 400 and 500 nM of nanocurcumin were selected for the study. Immediately before 6-OHDA application, it was diluted in 0.1% ascorbic acid and added to fresh cell culture medium to reach the required concentration (i.e. 50 µM according to previous reports (Amiri et al., 2016 ▶) and our pilot studies). Nanocurcumine was dissolved in DMSO at a stock concentration in which the final concentration of DMSO in culture medium did not exceed 0.1%. 

**Cell viability assay**

The cells cultured in 96-well plates at a density of 1×10^4^ cells/well, were treated the day after with 6-OHDA, with or without nanocurcumin. After 24 hr, the media was changed with the one containing 0.5 mg/ml MTT and incubated for 4 hr. Then, the media was gently removed and the precipitations in each well were dissolved in 100 μl of DMSO. The absorbance at 570 nm was measured using a microplate reader (Synergy HT, Biotek®).

**Cell morphology**

The morphological changes of the cells were assessed 24 hr after treatments. The cell numbers, cell body volume and debris were compared among groups. 

**Western blot analysis**

The cells were plated at the density of 10^6^ cell /cm^2 ^in 6-well plates. After 24 hr of treatments, SH-SY5Y cells were washed with ice-cold PBS and harvested using cell scraper in PBS. It was centrifuged at 1000 rpm for 5 min. The pellet was brought up in cold RIPA (Radio-Immunoprecipitation Assay) lysis buffer containing protease and phosphatase inhibitor cocktail. The cells were homogenized and allowed to maintain on ice for an additional 30 min. The cell lysates were centrifuged at 14,000 rpm for 30 min at 4°C to remove debris. The protein content of supernatants was determined by Lowry method and accordingly, the samples with equal amounts of protein (40 µg/ well) were separated using 10 % polyacrylamide gel electrophoresis and transferred to PVDF (polyvinylidene fluoride) membranes. After blocking in 5% BSA, the membranes were probed with primary antibodies (p-Akt, t-Akt and β-actin) overnight at 4 °C. The membranes were incubated for 2 hr at room temperature with horseradish peroxidase-conjugated anti-rabbit antibody. The blots were then revealed by ECL (Enhanced chemiluminescence) select kit. Finally the radiographic films were scanned and the density of bands was calculated by Image-J software. 

**Statistical analysis**

The MTT experiments and western blot analysis were repeated three times. The data was analyzed by one way ANOVA followed by Tukey as the *post hoc* test. In all statistical comparisons, a p<0.05 was considered significant.

## Results

**Characterization of antigen-loaded nanoparticles**

**Figure 1:**
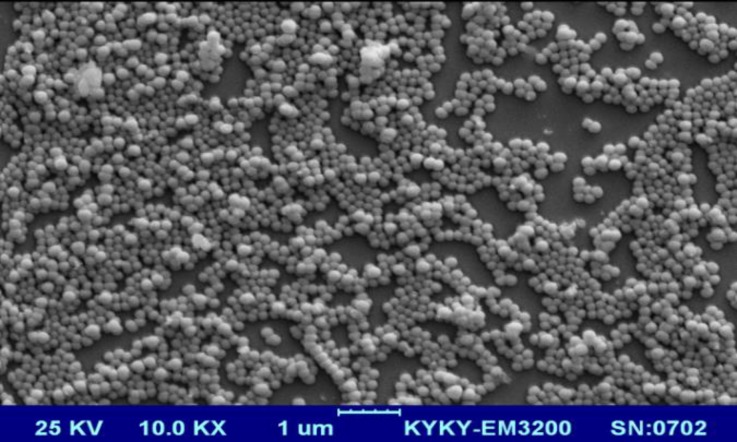
SEM images of the curcumin/BSA nanoparticles at different magnifications

SEM images (Figure 1) at different magnifications indicated formation of the nanoparticles by the desolvation method resulting in relatively regular-shaped spherical appearance and a smooth surface. Based on the results, a mean diameter of 153±20 nm was obtained for the nanoparticles.

**Figure 2:**
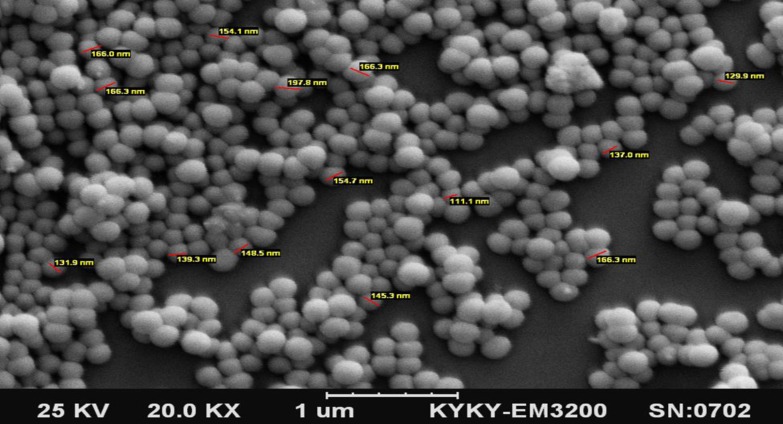
The effect of different doses of nanocurcumin against 6-OHDA-induced cell death in MTT assay (A). The selected doses of 400 and 500 nM of curcumin with/without 6-OHDA are compared with control (B). *p<0.05 represents signifcant difference between control and 6-OHDA group

**The effect of nanocurcumine against 6-OHDA-induced SH-SY5Y cell death**

A dose-response test was performed to assess if nanocurcumin protects against 6-OHDA toxicity. The results shown in Figure 2A, revealed that nanocurcumin at doses of 400 and 500 nM protects against 6-OHDA toxicity. Then, 400 and 500 nM of nanocurcumin were selected for further studies. The effect of nanocurcumine 400 and 500 nM with/without 6-OHDA on cell viability, is illustrated in Figure 2B. One-way ANOVA revealed a significant difference between groups (p=0.0088, F (5, 12) = 5.242). Also, *post hoc* analysis by Tukey test revealed that nanocurcumin 400 and 500 nM prevented 6-OHDA-induced cell death. Nanocurcumin by itself had no effect on cell survival comparing to the control group. To compare the effect of nanocurcumin with natural curcumin, another set of studies was done to explore the effect of curcumin on 6-OHDA-induced cell death. These results showed that curcumin is protective at the doses of 2 and 2.5 µM (data not shown). This means that BSA-based nanocurcumin is almost 4 times more potent than natural curcumin.

**Figure 3:**
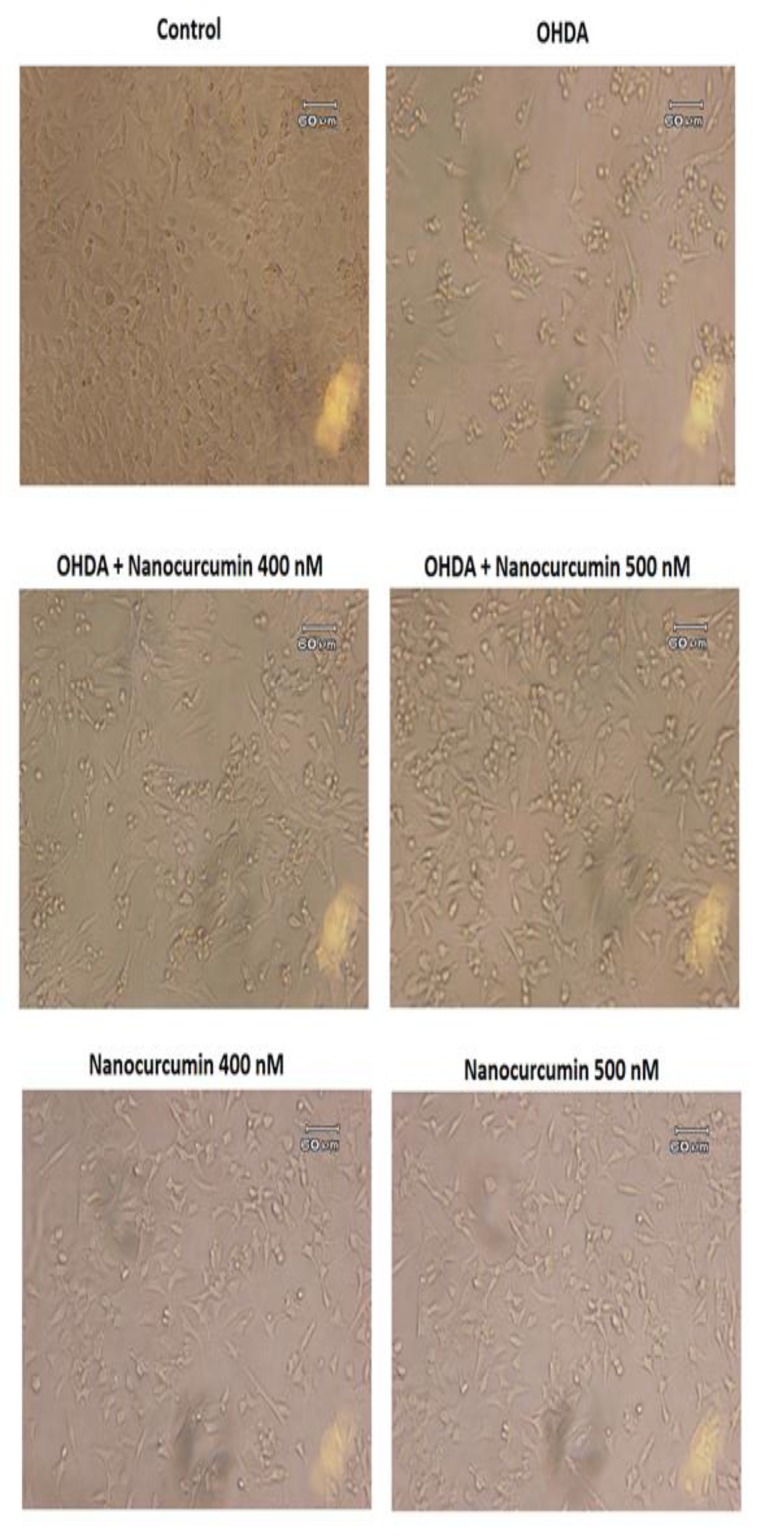
The microscopic images of SH-SY5Y cells in different groups. Images are magnified 20 times

**Changes of cell morphology**

The morphological results are shown in Figure 3. The pictures have been captured 24 hr after treatment. As shown in Figure 3, 6-OHDA lead to cell death, while nanocurcumin 400 and 500 nM protected the cells. There was no difference between nanocurcumin and control groups. 

**Western blot results**

**Figure 4:**
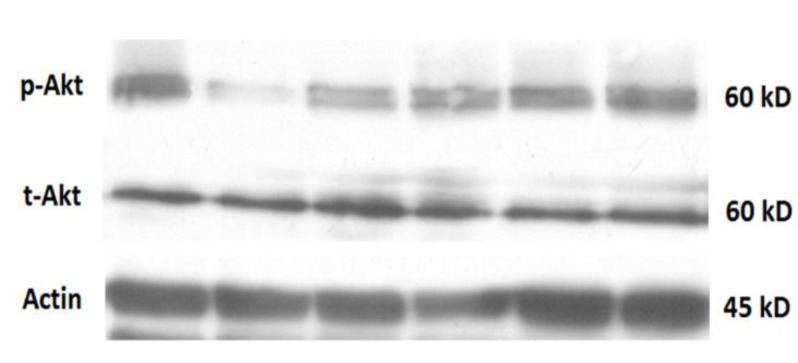
A) Western blot analysis showing p-Akt, t-Akt and actin contents in SH-SY5Y cells of different groups. B) p-Akt/t-Akt ratio in different groups. **p<0.01 represents a significant difference between control and 6-OHDA-treated cells

Western blot images showing the representative amounts of p-Akt, t-Akt and β-actin are shown in Figure 4A. The antibody against p-Akt or t-Akt detected a band at 60 kDa and the ratios of p-Akt/t-Akt in different groups are shown in Figure 4B. One-way ANOVA showed significant differences between groups (p value=0.0039, F (5, 12) = 6.450). Also, Tukey’s* post hoc* test revealed that 6-OHDA treatment decreased p-Akt/t-Akt ratio, while nanocurcumin 400 and 500 nM reversed this decrement.

## Discussion

Oral administration of drugs is assumed as the most convenient way for their delivery to the body (Rein et al., 2013 ▶; Scheepens et al., 2010 ▶) . Howbeit in neurodegenerative diseases such as PD, the existence of blood brain barrier (BBB) with a narrow diameter limits the transfer of materials especially for substances such as curcumin which is highly hydrophobic and has a poor absorption from the GI tract (Li et al., 2005 ▶) . Therefore, nanotechnology is used to break the molecules into nano-sized particles and increase their bioavailability and transfer through BBB (Tsai et al., 2011b ▶) . Albumin nanoparticles are commonly used as the carrier because of their high binding potency and non-toxicity. Desolvation, which is a method in which organic solvents are added to albumin aqueous solution followed by chemical cross-linking by glutaraldehyde, had been used previously to build BSA-based nanocurcumin (Aniesrani Delfiya et al., 2016 ▶; Jithan et al., 2011 ▶); this method was used in this study with some modifications. The SEM images confirmed that the particles had an average size of 153 ± 20 nm. This BSA-based nanocurcumin prevented 6-OHDA-induced cell death in human neuroblastoma cells as an *in vitro *model of PD (Song et al., 2012 ▶) . Comparing to natural curcumin, this nanoformulation was 4 times more potent in reversing cell viability which might be related to its easier transfer into the cells due to its smaller size. 6-OHDA is one of the metabolites of dopamine which has the capability to enter the cells through dopamine reuptake transporters (Ljungdahl et al., 1971 ▶) . It is suggested that this metabolite generates intracellular ROS and oxidative stress which ultimately lead to cell death (Hwang, 2013 ▶) . This condition resembles PD states as oxidative stress is supposed to play an important role in PD (Gu et al., 1998 ▶) . Curcumin has been known to function as an antioxidative substance with at least 10 times higher potency than vitamin E (Khopde et al., 2000 ▶) ; thus, the protective effect of nanocurcumin might be attributed to its antioxidative effect. Consistently, the protective effect of curcumin against paraquat in SH-SY5Y cells, has been shown to be mediated through modulating oxidative stress (Jaroonwitchawan et al., 2017 ▶) . 

The serine–threonine kinase Akt, which is also called PKB (protein kinase B), has been suggested to have a main role in cell survival (Datta et al., 1999 ▶; Franke and Cantley, 1997 ▶; Ghasemi et al., 2015 ▶) . Meanwhile, there are some evidence indicating that Akt is not always protective as its strong Akt activation has been shown to increase oxidative stress and induce cell death (Nogueira et al. 2008 ▶) . In PD patients, a decreased amount of striatal p-Akt has been reported (Greene et al., 2011 ▶) . In addition, 6-OHDA treatment of SH-SY5Y cells results in reduction of Akt phosphorylation  (Amiri et al., 2016 ▶; Chen et al., 2004 ▶; Driver et al., 2009 ▶**)** ; the finding which is in line with the results of the present study. These results indicate that Akt signaling is involved in human neuroblastoma cell survival which is compatible with some previous reports (Amiri et al., 2016 ▶; Chen et al., 2004 ▶; Driver et al., 2009 ▶) . This study also showed that the protective doses of BSA-based nanocurcumin corrected p-Akt/t-Akt signaling meaning that nanocurcumin reversed the effect of 6-OHDA on Akt signaling. Together, it is assumed that Akt has a role in the protective effect of nanocurcumin. 

In conclusion, this study showed that BSA-based nanocurcumin has a 4-time higher potency compared to natural curcumin in restoration of 6-OHDA-induced cell death in SH-SY5Y cells. Additionally, it seems that Akt signaling has a role in this protective effect. Considering the low bioavailability of curcumin, the BSA-based nanocurcumin might be suggested as a substitute for natural curcumin in treating neurodegenerative diseases.
